# Characterization of tumor-associated B-cell subsets in patients with colorectal cancer

**DOI:** 10.18632/oncotarget.1701

**Published:** 2014-05-09

**Authors:** Alexander Shimabukuro-Vornhagen, Hans A. Schlößer, Luise Gryschok, Joke Malcher, Kerstin Wennhold, Maria Garcia-Marquez, Till Herbold, Laura S. Neuhaus, Hans J. Becker, Anne Fiedler, Pascal Scherwitz, Thomas Koslowsky, Roland Hake, Dirk L. Stippel, Arnulf H. Hölscher, Sebastian Eidt, Michael Hallek, Sebastian Theurich, Michael S. von Bergwelt-Baildon

**Affiliations:** ^1^ Cologne Interventional Immunology, University of Cologne, Germany; ^2^ Department I of Internal Medicine, University of Cologne, Cologne, Germany; ^3^ Department of General, Visceral and Cancer Surgery, University of Cologne, Germany; ^4^ Department of Surgery, Marien Hospital, Brühl, Germany; ^5^ Department of Surgery, St. Elisabeth Hospital, Cologne, Germany; ^6^ Institute of Pathology, St. Elisabeth Hospital, Cologne, Germany

## Abstract

**Purpose:**

A precise understanding of the mechanisms by which human immune cell subsets affect tumor biology will be critical for successful treatment of cancer using immunotherapeutic approaches. Recent evidence suggests that B cells can both promote and inhibit the development and progression of tumors. The aim of this study was to characterize the composition of the B-cell infiltrates in colorectal cancers (CRC) in order to gain further insight into the role of B cells in CRC.

**Experimental Design:**

In this study we characterized B-cell subsets in primary tumors (n=38), metastases (n=6) and blood (n=46) of 51 patients with a diagnosis of CRC and blood of 10 healthy controls. B-cell subsets were analyzed by flow cytometry or immunohistochemistry.

**Results:**

Peripheral blood of CRC patients contained a higher percentage of memory B cells than that of age-matched healthy controls. Furthermore, the percentage of B cells within tumors was higher than that in the peripheral blood of CRC patients while metastases were typically devoid of tumor-infiltrating B cells. Tumor-associated B cells were enriched for activated and terminally differentiated B cells. Relevant proportions of regulatory B cells could only be detected in advanced cancer and metastases.

**Conclusion:**

B cells constitute a significant proportion of the immune infiltrate in CRC. The B-cell infiltrate of primary CRC is characterized by an accumulation of terminally differentiated memory B cells or plasma cells suggestive of a specific immune response against the tumor. However advanced tumors and metastases are also infiltrated by a considerable number of regulatory B cells.

## INTRODUCTION

The immune system plays an important role in the development and progression of cancer [1]. Immune cells, including T lymphocytes, macrophages, mast cells, and neutrophils present in the tumor microenvironment can either inhibit or enhance tumor growth. Little is known about the impact of B cells on tumor biology. The presence of B cells in human tumors has long been overlooked since the prevailing notion was that antitumor immunity is primarily mediated by T cells and NK cells. Since B cells were solely viewed as antibody producers and antibodies were believed to play a negligible role in tumor immunity their relevance in cancer biology has been ignored. In recent years, it has been demonstrated that B cells do also play an important role in tumor immunology [2]. However, the contribution of B cells to tumor immunology appears to be complex and entails both protumorigenic and antitumor effects. Experimental models have yielded important insights into the mechanism by which B cells affect tumor immunity. Besides antibody-mediated effects, antibody-independent mechanisms such as antigen-presentation [3], cytokine production [4], direct cytotoxicity [5] and indirect effects through modulation of other immune cells have been implicated to be of importance [6]. Whether B cells promote or inhibit tumor growth seems to depend on a number of variables such as temporal and spatial setting as well as on the composition of B-cell subsets. The findings in murine tumor models raised renewed interest in studying the B-cell infiltrate in human tumor samples and its potential impact on the tumor microenvironment. Indeed, B-cell infiltrates can be found in many different human tumor entities, including breast cancer [7], lung cancer [8], ovarian cancer [9], colorectal cancer [10] and germ cell tumors [11].

The multitude of B-cell-directed agents which are on the market or in development, predominantly for the treatment of autoimmune diseases and B-cell malignancies, offer the perspective that insights into the role of B cells in human tumor biology can be rapidly translated into clinical interventions. A more detailed understanding of tumor-associated B-cell subsets and their effects on tumor growth is therefore crucial and will facilitate the therapeutic manipulation of the B-cell compartment with the aim of enhancing tumor immunity.

Since most studies to date used immunohistochemistry on paraffin-embedded tissues they could only assess a limited number of markers and an identification of specific B-cell subsets, which are defined by coexpression of multiple markers, was not possible. We thus set out to perform a comprehensive flow cytometric characterization of tumor-associated B cells in peripheral blood and fresh tumor samples of patients with colorectal cancer.

## RESULTS

### IgD^−^CD27^+^ memory B cells are increased in peripheral blood of CRC patients

**Figure 1:**
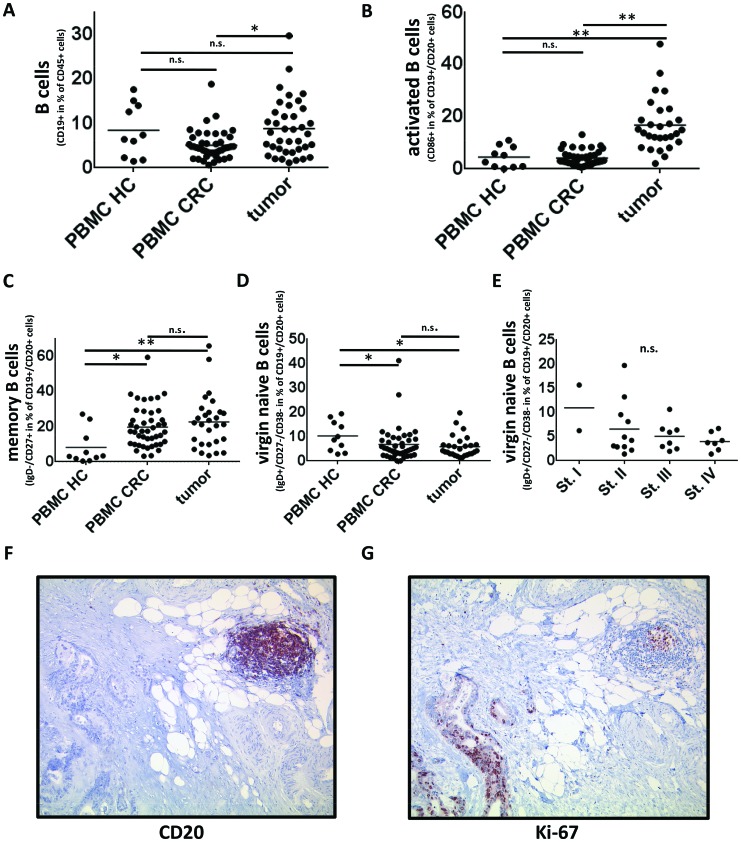
B cells and B-cell subsets in PBMC and tumor samples of CRC patients and PBMC of healthy controls **(A)** Flow cytometric analyzes of CD19^+^ cells in PBMCs of healthy controls (n=10), PBMCs of CRC patients (n=46) and single cell suspensions of tumor samples (n=38). The percentage of CD19^+^ cells in tumor samples is significantly higher than in PBMC of CRC patients (8.9% vs. 5.1% of CD45+ lymphocytic cells, p<0,05). **(B)** Tumor associated B cells (n=28) contain a higher percentage of activated B cells (defined by positivity for CD19, CD20 and CD86) than PBMCs of CRC patients (n=44) or healthy controls (n=10) (16.8% vs. 4.1/4.5% respectively, p<0,005). **(C)** CD27^+^IgD^−^ (memory) B cells are elevated in PBMCs (n=44) and tumor (n=28) of colorectal cancer patients compared to PBMCs of healthy controls (n=10) (19.3/22.7% vs. 8.2%, p<0,05/<0,005 respectively). **(D)** According to an increase in memory B cells virgin naïve (IgD+/CD27-/CD38-) B cells are decreased in PBMCs (n=44) and tumor samples (n=28) of CRC patients compared to PBMC of healthy controls (n=10) (p<0,05) **(E)** The percentage of IgD+/CD27-/CD38- naive B cells decreases with UICC stage (n=28, n.s.). **(F)** Exemplary immunohistochemical staining of CD20 to visualize the intratumoral distribution of tumor-associated B cells, predominantly in peritumorous lymphoid follicles. **(G)** Immunohistochemical analyzes of Ki-67 in CD20^+^ cells revealed a strong local proliferation of B cells within tertiary lympoid structures.

We assessed the composition of the B-cell populations in peripheral blood of 46 cancer patients and compared it to 10 age- and sex-matched healthy controls. The clinical characteristics of the patients are summarized in table 1 and the pathologic features are listed in supplementary table 1. The percentage of CD19^+^ B cells among CD45^+^ lymphocytes in the peripheral blood of colorectal cancer patients did not differ significantly from healthy controls (8.4% vs. 5.1%, p=0.14, Fig. 1A). Although the percentage of total circulating B cells was similar, the composition of the B cell subsets showed large differences. The percentage of memory B cells (defined as IgD^−^CD27^+^) in peripheral blood of CRC patients was more than twice as high (19.3% vs. 8%, p<0.005, Fig. 1C), suggesting an impact of CRC on B-cell homeostasis. Conversely, CD45^+^ PBMCs of healthy donors contained more virgin naïve (IgD^+^CD27^−^CD38^−^) B cells (p<0.05, Fig. 1D). An analysis of the correlation of B cells and B-cell subsets in peripheral blood of CRC patients with the UICC-tumor-node-metastasis-classification revealed similar compositions throughout the stages (data not shown). As shown in figure 1F and 1G there is a strong colocalisation of CD20 and Ki67 in sequential immunohistochemical stainings, suggesting a local proliferation of tumor associated B cells. This characteristic distribution of CD20 and Ki67 with strong Ki67-positivity especially in germinal centers of peritumorous tertiary lymphoid structures could be confirmed in >90% of the analyzed samples.

**Table 1:** Patient characteristics

**CRC patients (n=51)**	Age	70 (±11)	
	Sex	32 (61%) male	19 (39%) female
	UICC Stage	St. I	2 (4%)
		St. II	17 (34%)
		St. III	14 (27%)
		St. IV	18 (35%)
	Grading (n.d. in 4 Metastases)	G 1	0
		G2	41
		G3	6
	Primary site (RCTx)	34 Colon (0)	17 Rectum (1)
**Healthy donors (n=10)**	Age	65 (±11)	
	sex	4 (40%) male	6 (60%) female

### Tumor-associated B cells are activated and of a memory phenotype

**Figure 2:**
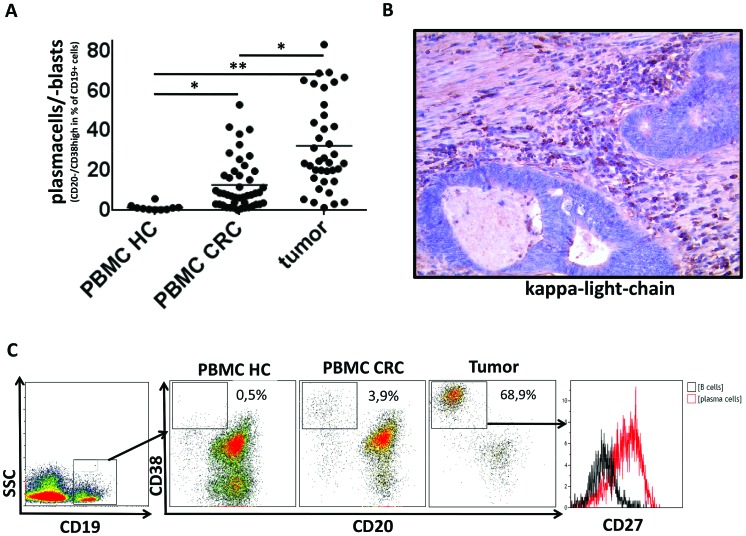
Plasma cells in PBMC and tumor samples of CRC patients and PBMC of healthy controls **(A)** Plasma cells (CD19^+^CD20^−^CD38^high^) are strongly elevated in PBMCs (n=46) and tumor tissue (n=38) of CRC patients compared to healthy controls (n=10) (12.6%/31.7% vs. 1.4% respectively; p<0,05/<0,005) **(B)** Immunohistochemistry showing an infiltration of colorectal cancer by IgG-kappa^+^ cells into the same tumor sample as shown in C. **(C)** Exemplary density plots showing CD45^+^CD19^+^CD20^−^CD38^high^ cells in peripheral blood and tumor tissue of CRC patients and peripheral blood of healthy controls. The plasma cell phenotype of these cells was confirmed by expression of CD27.

Single cell suspensions of tumor tissue of 38 CRC patients were produced within 12 hours after surgery. Subsequently we analyzed the tumor-infiltrating lymphocytes by 10-color flow cytometry. CD19^+^ cells could be detected in all 38 tumor samples and on average made up 8.9% of the CD45^+^ lymphocytic infiltrate of colorectal cancers compared to 5.1% of the CD45^+^ PBMCs of CRC patients (p<0,005, Fig. 1A). Of note, about one third (31.7%) of the tumor-infiltrating CD19^+^ cells were CD19^+^CD20^−^CD38^high^ plasma cells (Fig. 2A). CD3^+^ T cells were also detectable and made up 68% of the CD45^+^ lymphocytic infiltrate (data not shown). A well-defined population of cells co-expressing CD19^+^ and CD20^+^ could be detected in 28 out of 38 tumor samples with a non-significant increase in metastatic disease (8.3% in stage IV vs. 6.4% in stage I–III, p=0.28). In further analyses of these samples tumor-associated B cells have shown several differences to B cells in the peripheral blood of CRC patients and healthy controls. Tumor samples revealed a higher percentage of activated B cells (characterized by expression of CD19, CD20 and CD86) than PBMCs of CRC patients (16.8% vs. 4.1%, p <0,005, Fig. 1B) or healthy controls (16,8% vs. 4,5%, p<0,005, Fig. 1B). To assess the maturity of B cells in the analyzed samples virgin naïve (CD19^+^CD20^+^IgD^+^CD27^−^CD38^−^), virgin activated (CD19^+^CD20^+^IgD^+^CD27^+^ CD38^−^) and memory B cells (CD19^+^CD20^+^IgD^−^CD27^+^) were quantified. Whereas memory B cells were relatively rare in peripheral blood of healthy controls (8.2%) they made up 22.7% of CD19^+^CD20^+^ cells in tumor samples (p<0,005, Fig.1C). The percentage of memory B cells was similar across all UICC stages (data not shown). According to these results virgin naive B cells were higher in PBMCs of healthy controls compared to PBMCs and tumor tissue of CRC patients (10.2% vs. 6.8%, 6.3% respectively, p<0.05; Fig. 1D). The percentage of virgin naïve B cells in tumor samples showed a tendency to decrease with higher UICC stages (Fig. 1E, n.s.). Virgin activated B cells were hardly detectable in tumor samples and their percentage was similar in PBMCs of tumor patients and healthy controls (data not shown).

### Plasma cells are elevated in peripheral blood and tumor tissue of colorectal cancer patients

Analysis of plasma cells revealed marked differences between cancer patients and healthy controls. Plasma cells were rarely detectable in PBMCs of healthy donors (n=10) but made up a significant part of CD19^+^ lymphocytes in peripheral blood (n=46) and tumor tissue (n=38) of colorectal cancer patients. The percentage of CD19^+^CD20^−^CD38^high^ cells was 1.4% of CD19^+^ lymphocytes in PBMCs of healthy controls compared to 12.6% in PBMCs and 31.7% in the tumor tissue of CRC patients (p<0,005, Fig.2A). The plasma cell phenotype was confirmed by a strong expression of CD27 in CD19^+^CD20^−^CD38^high^ cells (Fig. 2C). Additionally, the results obtained by flow cytometry were confirmed by immunohistochemical staining for IgG-kappa (Fig. 2B). Nephelometry of IgG subclasses in 11 accessible serum samples of CRC patients revealed a normal distribution of IgG subclasses (IgG1>IgG2>IgG3>IgG4) in 9/11 cases (data not shown).

### B-cell subsets with a regulatory phenotype (CD24^high^CD38^high^ and CD24^high^CD27^+^) are detectable in tumor samples and peripheral blood of CRC patients

**Figure 3:**
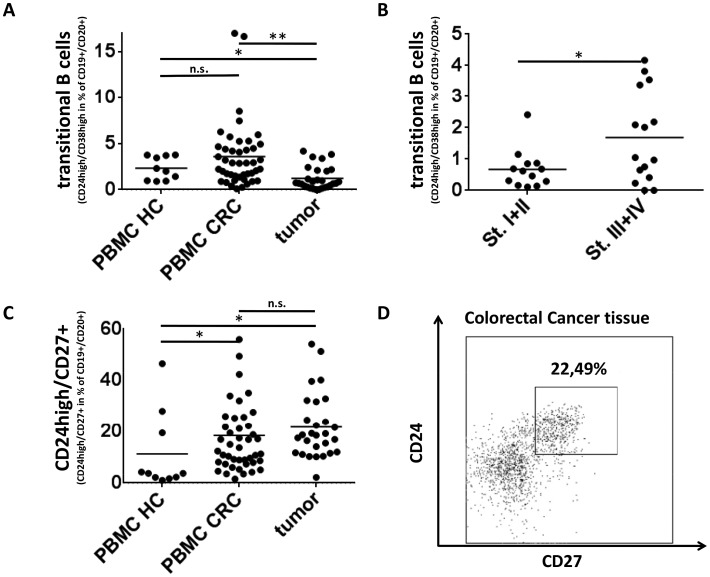
Regulatory B-cell subsets in Colorectal Cancer **(A)** The percentage of CD24^high^CD38^high^ (transitional) B cells is lower in tumor samples (n=28) than in peripheral blood of CRC patients (n=44) or healthy controls (n=10) (1.2% vs. 3.5%/2,3%, p<0,005/p<0,05 respectively). **(B)** Whereas transitional B cells are very rare in early stage CRC the frequency increases in advanced stage disease (n=28) (0.7% of B cells in Stage I+II vs. 1.8% in Stage III+IV, p<0,05). **(C)** CD24^high^CD27^+^ B cells make up a high percentage of tumor associated B cells in tumor samples (n=28) and PBMC of colorectal cancer (n=44) and are significantly elevated compared to PBMC of healthy controls (n=10) (21.1%/18.5% vs. 11.3%, p<0,05). **(D)** Exemplary density plot showing CD24^high^CD27^+^ B cells in a tumor sample from a patient with colorectal cancer (gated on lymphocytic FSC/SSC,CD45^+^,CD19^+^,CD20^+^).

A regulatory function has been described for transitional B cells (CD19^+^CD20^+^CD24^high^CD38^high^, Fig. 3A). Our analysis of this subset revealed no difference between PBMCs of CRC patients and healthy donors. Although the frequency of transitional B cells in tumor tissue was lower than that in peripheral blood of CRC patients (1.2% vs. 3.5%, p<0,005) transitional B cells were detectable in tumor samples and their percentage was increased in advanced stage disease (0.7% of B cells in Stage I+II vs. 1,8% in Stage III+IV, p<0,05, Fig.3B). The CD24^high^CD27^+^ B cell population has recently been described as another regulatory B cell subset [12]. This population was regularly detected within tumor-associated B cells and PBMCs of CRC patients and was significantly elevated compared to PBMCs of healthy controls (21.1%/18.5% vs. 11.3%, p<0,05, Fig. 3C,D). The percentage of CD24^high^CD27^+^ B cells in tumor samples did not correlate with tumor stage (data not shown).

### Characterization of B cell subsets in colorectal liver metastases

**Figure 4:**
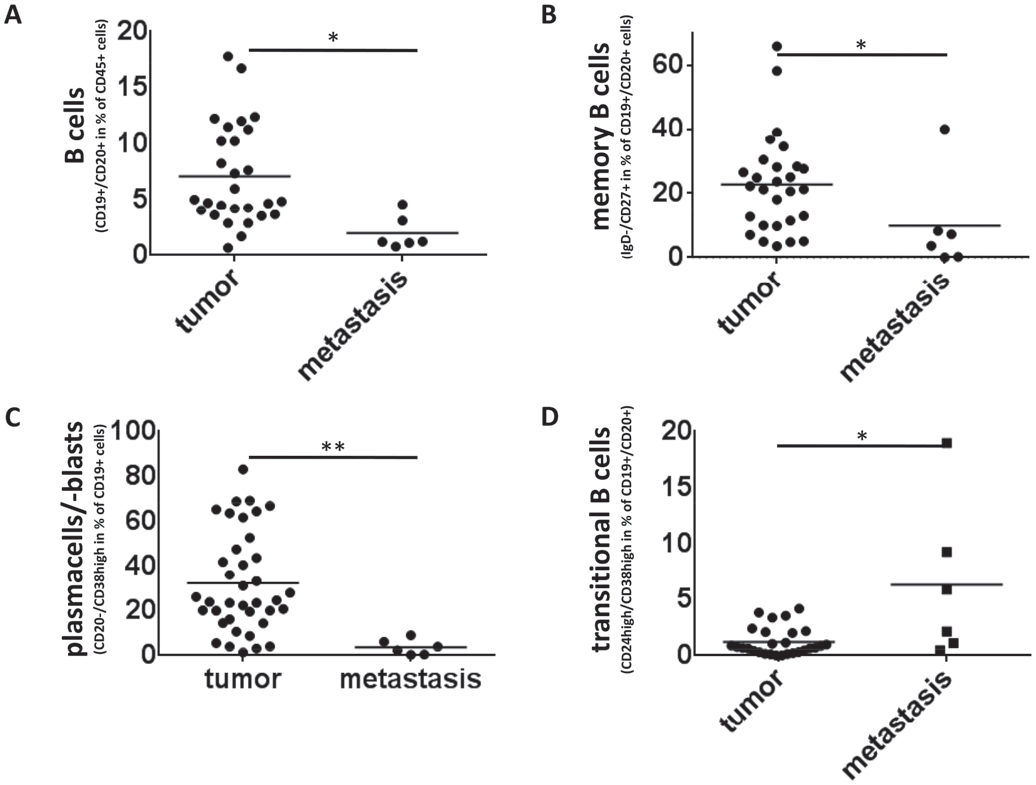
Memory B cells and cells with a plasma cell phenotype are significantly reduced in colorectal liver metastasis **(A)** CD19^+^CD20^+^ cells make up only 2% of CD45+ lymphocytic cells in metastatic tissue (n=6) compared to 6.9% in samples of primary colorectal cancer (n=28) (p<0,05) **(B)** The percentage of IgD-/CD27+ memory b cells in colorectal liver metastasis (n=6) is significantly lower than in primary tumors (n=28) (9.9% vs. 23.4%, p<0,05) **(C)** Whereas cells with a plasma cell phenotype were regularly detectable in tumor samples of CRC patients (n=38) they were heavily reduced in metastatic Tissue (n=6) (3.7% compared to 31.7%, p<0,005). **(D)** B cells with a regulatory phenotype (CD24^high^CD38^high^ B cells) were rare in the primary tumors, but well detectable in metastatic tissue (1,1% vs. 6,3% of CD19^+^CD20^+^ cells, p<0,05).

To assess differences between infiltrating lymphocytes in metastatic tumor tissue and samples of the primary tumor of colorectal cancer patients, biopsy specimens of six colorectal liver metastases were processed and stained analogous to the primary tumors. Our analyses showed a lower percentage of CD19^+^CD20^+^ cells in metastatic samples than in primary tumors (2% vs. 6,9% of CD45^+^ lymphocytic cells, p<0,05, Fig. 4A). Analyses of the state of activation and maturity revealed a lower percentage of memory B cells (IgD^−^CD27^+^) in metastatic tissue than in primary tumors (23.4% vs. 9.9% of CD45^+^CD19^+^CD20^+^ cells, p<0,05, Fig. 4B). Whereas most primary tumor samples contained a distinct plasma cell infiltrate, CD19^+^CD20^−^CD38^high^ cells were very rare in metastatic samples (31.7% vs. 3.7% of CD45^+^CD19^+^ cells, p<0,005, Fig. 4C). Interestingly CD24^high^CD38^high^ “transitional” B cells, which represent a regulatory B cell subset, were of low frequency in the primary tumors, but a significant fraction of the metastatic tissue (1,1% vs. 6,3% of CD19^+^CD20^+^ cells, p<0,05, Fig. 4D).

### Tumor-infiltrating T lymphocytes are mainly CD45^−^CCR7^−^ effector/memory T cells and CD3^+^CD4^+^CD25^+^CD127^low^ regulatory T cells are significantly elevated in tumor samples

**Figure 5:**
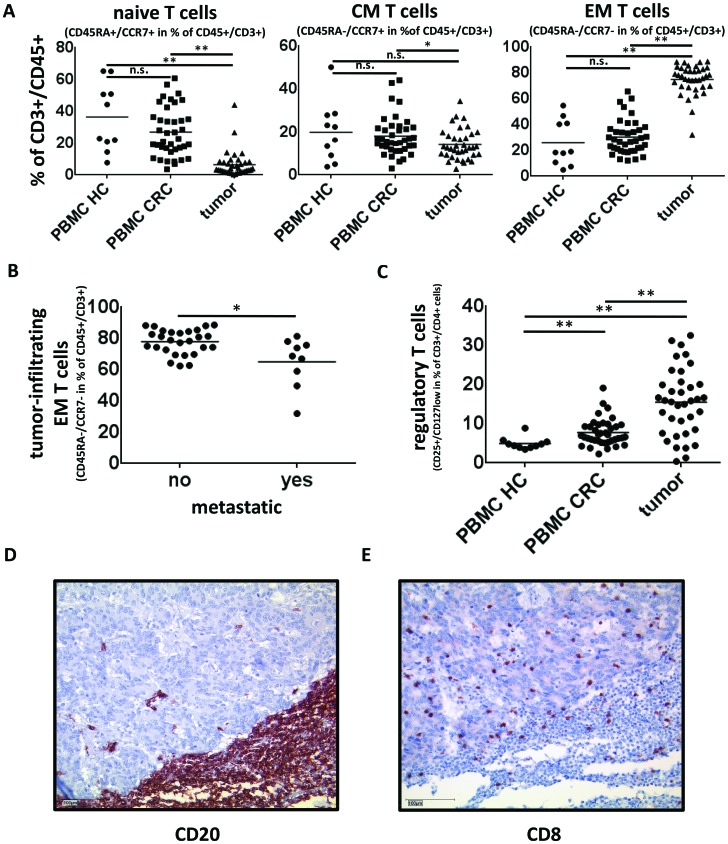
Tumor infiltrating T cells in colorectal cancer **(A)** Tumor infiltrating T cells are mainly of a CD45RA^−^CCR7^−^ effector/memory (EM) phenotype (n=37) whereas PBMC of CRC patients (n=38) and healthy controls (n=10) contain predominantly naive (CD45RA^+^CCR7^+^) and central memory (CM) (CD45RA^−^CCR7^+^) T cells. **(B)** The percentage of CD45RA^−^CCR7^−^ effector memory T cells in primary tumor samples is significantly reduced in Stage IV colorectal cancer (65% vs. 77,9%, p<0,05) **(C)** CD3^+^CD4^+^CD25^+^CD127^low^ T cells which are known to contain a high percentage of FoxP3+ regulatory T cells are significantly elevated in tumor samples (n=38) compared to PBMC of CRC patients (n=39) and healthy controls (n=10) (15.5% of CD4^+^-Tcells in tumor samples compared to 7.8% and 5.0% respectively, p<0,005) (**D,E**) Immunohistochemical staining of CD20 and CD8 were performed to illustrate the intratumoral distribution of B and T cells. B cells were detectable throughout the whole tumor samples, but were predominantly localized in peritumoral tertiary lymphoid structures. Figure E highlights the infiltration of Colorectal Cancer by cytotoxic T cells and also demonstrates a colocalisation of cytotoxic T and B cells especially within the tertiary lymphoid structures. Sequential immunohistochemistries of CD8^+^-T cells and CD20^+^-B cells revealed similar colocalisations in 40% of the analyzed samples.

Whereas naive T cells (CD45RA^+^CCR7^+^) make up about 25% of T cells in the peripheral blood of CRC patients and healthy controls their percentage in tumor samples is less than 10%. Tumor-infiltrating T cells are predominantly antigen experienced CD45^−^ CCR7^−^ effector/memory T cells (74.7% of CD45^+^CD3^+^ lymphocytic cells in tumor tissue compared to 30.2% and 26% in PBMCs of CRC patients and healthy controls respectively, p<0,005, Fig. 5A). Of note, the percentage of effector/memory T cells decreases in metastatic disease (Fig. 5B). In order to assess regulatory T cells we analyzed the CD3^+^CD4^+^CD25^+^CD127^low^ T cell subset, which is known to contain a high percentage of FoxP3^+^ T_reg_. CD3^+^CD4^+^CD25^+^CD127^low^ T cells made up 15.5% of CD4^+^-T cells in tumor samples compared to 7.8% and 5.0% of PBMCs of CRC patients or healthy controls respectively (p<0,005, Fig. 5C). Since regulatory B cells have been proposed to expand regulatory T cells we investigated whether the amount of regulatory B cells and regulatory T cells in the tumor correlated [13,14]. Pearson's test revealed no significant correlation between CD3^+^CD4^+^CD25^+^CD127^low^ regulatory T cells and either the CD24^high^CD38^high^ (r=0,283) or the CD24^high^CD27^+^ (r=0,183) B-cell subsets. Cytotoxic CD8^+^ T lymphocytes were present both within and around the tumor while B cells clustered primarily at the invasive margins (Fig. 5D,E). The B cells frequently formed follicle-like structures along the tumor border.

## DISCUSSION

The lymphocytic infiltrate of tumors is known to have an important impact on the prognosis of malignant diseases [15–17]. In colorectal cancers a classification based on the immune infiltrate seems to be superior to conventional tumor staging [18]. Although this and other studies focused on T cells, B cells have also been shown to be an important part of the immune cell infiltrate of many tumor entities [2]. However, the role of B cells in tumor development and immunosurveillance is complex [19]. On the one hand, data from animal models suggest that B cells seem to support tumor development and survival. Murine tumor models have provided crucial information about the underlying mechanisms of B-cell mediated effects on tumor biology. Antibodies produced by B lymphocytes participate in carcinogenesis and tumor progression by promoting chronic inflammation [20]. Furthermore, regulatory B cells have been shown to suppress T cell-mediated antitumor responses [21–23]. In line with these results depletion of B cells enhances T cell immunity in response to cancer vaccines [24,25]. In addition to promoting the development and growth of tumors, B lymphocytes can also enhance cancer metastasis [13,26,27].

On the other hand, studies have shown that tumor-associated B cells also contribute to cancer immunosurveillance. B cells are required for optimal CD4 and CD8 T cell tumor immunity [3]. Conversely, depletion of B cells in this setting promotes tumor progression. In addition, B-cell mediated antitumor immunity can also suppress cancer metastasis [28]. Immunohistochemical studies of human cancers found that infiltration of the primary tumor with B cells correlate with a better prognosis and lower rate of metastasis [29,30]. In addition, B-cell based cancer immunotherapy in animal models has yielded promising results and demonstrates that B cells can induce T-cell mediated antitumor immunity [31–34]. How B cells exert their antitumor effects is not precisely known. In some settings the antitumor activity was primarily antibody-mediated [35] whereas other studies suggested a crucial role for antigen-presentation by B cells [36]. Interestingly, a recent retrospective analysis of lymphoma patients receiving high-dose chemotherapy with subsequent autologous transplantation showed that the addition of the B-cell depleting monoclonal anti-CD20 antibody, rituximab, to a high-dose chemotherapy regimen resulted in a significantly higher incidence of solid tumors from 4.90% to 13.26% after ten years of follow-up, suggesting that in humans B cells contribute to tumor immunosurveillance [37].

The heterogeneity of tumor-infiltrating B-cell subsets could provide an explanation as to why studies on B cell-depletion in cancer found both antitumorigenic or protumorigenic effects [38,39]. Understanding the composition of B cell-infiltrates in human tumors is therefore crucial for the effective treatment of cancer patients with B cell-targeted therapies. Several studies have investigated tumor-infiltrating B cells in human malignancies using immunohistochemistry or transcriptomic analysis [9,40,41]. They found that a B-cell infiltrate is present in a wide variety of human tumor entities and that the presence of B cells correlates with survival. Due to the technical limitations of immunohistochemistry and gene expression analysis these studies could not provide a detailed characterization of the different subsets within tumor-associated B cells. We thus used 10-color flow cytometry to perform a more in depth phenotypic analysis of B cells from peripheral blood and tumor samples of patients with colorectal cancer. Using flow cytometry we could identify several B-cell subsets, which are defined by up to five different markers (e.g. transitional B cells (CD45^+^,CD19^+^,CD20^+^,CD24^high^,CD38^high^). The use of flow cytometry to study the composition of the intratumoral B-cell compartment enabled us to gain more detailed information on B cell subtypes than would be feasible by immunohistochemical methods.

Since the importance of the immune response has been well established in colorectal cancer this entity appears to be an attractive model to study the impact of B cells on the clinical course of solid cancers in humans. [18]. A small case series reported a beneficial effect of B-cell depletion in patients with colorectal cancer suggesting that therapeutic targeting of B cells could be a promising treatment approach in CRC patients [38]. Many solid tumors induce a disturbance of the B cell compartment. In a recent study which investigated B cells in the peripheral blood of cancer patients, cancer progression lead to a disturbance of B-cell homeostasis and resulted in a loss of memory B cells as well as an increase in plasmablasts in the peripheral blood of cancer patients [42]. Regarding peripheral blood, our results were similar with a remarkable increase of cells with a CD19^+^CD20^−^CD38^high^CD27^+^ plasma cell phenotype. Of note, we could also detect the same population within colorectal cancer tissues, confirming immunohistochemical studies showing a pronounced plasma cell infiltration in colorectal cancer [43] and other tumor entities [9,44,45]. In colorectal cancer a plasma cell infiltrate is associated with favorable outcome [43,46]. Furthermore, several studies yielded indirect evidence that tumor-infiltrating B cells in some solid tumors contain a high proportion of tumor antigen-specific B cells. Analysis of the immunoglobulin genes from tumor-infiltrating B cells revealed a restricted repertoire with a high frequency of B cells specific for tumor-associated antigens [47,48]. Our analyses of IgG-subclasses in 11 accessible serum samples showed a normal distribution of IgG subclasses suggesting that the peripheral antibody repertoire is not significantly disturbed. However, we could not assess whether the intratumoral antibody repertoire was affected.

In comparison to the published literature on B cells in solid tumors our study in CRC patients reveals some commonalities but also important differences. Similar to the results described by Carpenter et al we found an increase of plasma cells in peripheral blood of cancer patients with advanced cancer [42]. Concerning the activation status of B cells, they found no significant difference in the percentage of CD86^+^ cells (6.7% vs. 4.2%) between tumor patients and controls. This is in line with our results for CRC patients. With regard to other B-cell populations our results differ from those of Carpenter et al [42]. In their report the percentage of circulating CD19^+^ cells was increased in malignant melanoma patients, whereas our study shows a significant decrease compared to healthy controls. They also describe a decrease in memory B cells with tumor progression. On the contrary, we observed an increased percentage of memory B cells in the peripheral blood of CRC patients. The differences between the results of Carpenter and our study can be due to a number of methodological differences between the two studies. The patients in the study of Carpenter et al consisted primarily of patients with malignant melanoma. Additionally, since the peripheral blood samples in our study were exclusively obtained prior to surgery, our study only included the subgroup with active disease as described by Carpenter et al. The different results concerning memory B cells of our study compared to the results of Carpenter et al in peripheral blood of malignant melanoma patients are most likely a result of differences in the gating strategy. We gated on CD19^+^CD20^+^ lymphocytic cells whereas Carpenter et al described the percentage of CD19^+^ cells. In addition, we included IgD-negativity to define CD27^+^ memory B cells. In line with our findings, the percentage of IgD^+^ cells was decreased in peripheral blood of patients with active malignant melanoma [42].

In addition to peripheral blood, we also analyzed B cells in freshly isolated and processed solid tumor samples of colorectal cancer patients. To ensure that samples comprised of representative tumor tissue the tumors were dissected by an experienced pathologist. To our knowledge, there are only two other studies addressing this issue: Nielsen et al analyzed tumor-associated B cells in ovarian cancer, including flow cytometric analyses of 9 tumor samples and Zirakzadeh et al. studied 7 urinary bladder cancer and 1 prostate cancer sample [9,40]. Our analysis revealed a highly activated B cell infiltrate. CD86^+^ activated B cells were significantly elevated in tumor tissue compared with PBMCs of healthy controls and CRC patients. This is in line with the results obtained in ovarian and urinary bladder cancer specimens using Ig-class-switch or CD69-positivity to characterize activated B cells [40]. In the study published by Nielsen et al. the majority of CD20^+^ B cells were IgG^+^ and classified as memory B cells with an unusual down-regulation of CD27. We observed a similar population (IgD^−^CD27^−^) but also a significant increase of classical IgD^−^CD27^+^ memory B cell subset suggesting that a down regulation of CD27 is less frequent in colorectal cancer than in ovarian cancer (Fig. 2B). Zirakzadeh et al also described an increased proportion of memory B cells in urinary bladder cancer. We visualized the distribution of tumor associated T and B cells by immunohistochemistry. Sequential stainings revealed a colocalization of Ki67 and CD20, suggesting a local proliferation, especially in peritumorous tertiary lymphoid structures. For T cells it is well established that tumor-infiltrating cells are mainly of an effector memory phenotype and our analyses confirmed this finding (Fig. 5A).

For both T and B cells an immunosuppressive/regulatory subset has been described with FoxP3^+^ regulatory T cells being the most well known subset. We observed a significant increase of regulatory T cells in colorectal cancer specimens thereby confirming prior studies [49]. Whereas regulatory T cell subsets have been extensively studied there is little knowledge about the role of regulatory B cells in human cancer [50]. To address this issue we included two recently described regulatory B-cell subsets, i.e. CD24^high^CD38^high^ and CD24^high^ CD27^+^B cells, in our analyses. CD24^high^CD38^high^ “transitional” B cells could be detected in most colorectal cancer samples, but their frequency was generally low in tumors and even lower in peripheral blood. Of interest, the percentage of CD24^high^CD38^high^ B cells was significantly elevated in advanced stage disease (Fig. 3A, B). Iwata et al. recently described CD24^high^CD27^+^ B cells as the major B cell population producing the immunosuppressive cytokine IL-10 in humans [12]. This population was present in the majority of tumor samples and in some cases represented more than 50% of CD19^+^CD20^+^ B cells (Fig.3 C,D). As we did not include IL-10 in our analyses, it needs to be further studied whether this population reflects the same IL-10 producing B cell subset. However, since we did not find a correlation between any of the two regulatory B cell subsets and the amount of regulatory T cells this finding would argue against a role of these regulatory B cell subsets in the expansion of regulatory T cells.

Since we observed changes in the proportion of the different B-cell subsets with disease progression we also analyzed biopsy specimens from liver metastases of six patients with metastatic colorectal cancer. Studies investigating the role of B cells in cancer metastasis have yielded conflicting results. Some studies found that a strong B cell infiltrate of the primary tumor was associated with a lower risk of distant metastasis [28,51] while others found the exact opposite [13,26,27,30,52]. We detected B cells in a significantly lower frequency than in primary tumor samples comprising less terminally differentiated B cells. IgD^+^CD27^−^ memory B cells were decreased from 23.4% in tumor tissue to 9.9% among CD19^+^CD20^+^ B cells in colorectal liver metastases. The absence of B cells in colorectal cancer metastases is in line with results in metastatic melanoma [53]. Contrary to primary tumors, where we saw a strong infiltration by cells with a plasma cell phenotype this subset made up only 3.7% of CD19^+^ cells in metastatic tissue. However, regulatory B cells were significantly increased in metastatic tissue indicating a shift in the balance of B-cell subsets towards a more immunosuppressive state. These findings may reflect an immune escape mechanism of metastatic tumor cells, which results in a decreased detection by and activation of B cells.

T follicular helper cells (Tfh) are a novel T cell subset. It has recently been shown that Tfh cells also contribute to tumor immunity and that there is a correlation between B cells and Tfh within the tumor microenvironment [41,54]. Due to the importance of the interaction between Tfh and B cells during the germinal center reaction it would be interesting to investigate the cooperation of both cell types within the tumor microenvironment. The first reports that implied a role of Tfh in tumor immunology were published after the samples of our study had been analyzed. Therefore Tfh were not included in our FACS panel. In the future we are planning to study both populations using flow cytometry to clearly identify Tfh by multi-parameter flow cytometric analysis.

One limitation of our study is the low number of samples. This resulted in a low statistical power and limited our ability to identify statistically significant differences between the immune cell subsets. Compared to immunohistochemistry or gene expression analysis the flow cytometric assessment of the tumor immune infiltrate is more expensive and more time-consuming. Nonetheless, our study represents the largest flow cytometric characterization of tumor-infiltrating B lymphocytes published so far. To our knowledge this is the first report providing a detailed phenotypic analysis of the B-cell infiltrate in colorectal cancer.

In summary, our results show that patients with colorectal cancer have substantial alterations in their B cell compartment. Tumor-associated B cells are predominantly activated and of a mature phenotype suggesting a specific response to the tumor. On the other hand, metastatic lesions are characterized by a low number of B cells and an increased proportion of regulatory B cells, which might reflect immune escape. Importantly, this study also demonstrates that the composition of immune cells subsets in peripheral blood reflects changes in the tumor microenvironment. Our results provide important information for B cell-targeted therapeutic interventions and suggest that B cell-depletion at early tumor stages would be detrimental whilst in metastatic disease B cell-depletion could potentially enhance antitumor immunity through the depletion of regulatory B cells.

## MATERIALS AND METHODS

### Patient characteristics

Tumor samples (n=38), peripheral blood mononuclear cells (PBMCs) (n=46) and metastatic tissue (n=6) of 51 patients with a diagnosis of colorectal cancer were obtained between August 2010 and January 2013. Tumor stage was assessed according to the UICC tumor-node-metastasis criteria. Peripheral blood samples of 10 healthy donors were included as controls for PBMCs. The patient characteristics are summarized in table 1. Written informed consent was obtained from all patients prior to surgery and our institutional review board approved the study.

### Cell isolation from human peripheral blood, tumor and metastatic tissue

Peripheral blood was obtained after written, informed consent from patients and age- matched healthy donors immediately prior to surgery. Peripheral blood mononuclear cells were purified using density-based separation with Ficoll-Paque PLUS (GE Healthcare Life Sciences).

Fresh unfixed tissue from primary tumor or metastatic lesions, which were not required for pathological analyses, were transferred to our laboratory immediately after surgical resection and processed within 12 hours. The samples were provided by an experienced pathologist (S. Eidt) who made sure that the samples we obtained were indeed tumor tissue. Furthermore histological analysis of the resection margins of our samples was performed. The fresh tumor tissue was manually minced using a scalpel and then transferred into single cells suspensions using a gentle MACS Dissociator (Miltenyi, Bergisch Gladbach, Germany). Single cell suspensions were obtained by sequential dissociation and incubation according to the company's instructions. Afterwards, the cells were filtered through 70 μm nylon cell strainers (BD).

### Flow cytometry

If possible at least 1x10^6^ events per sample were acquired on a Gallios 10-color flow cytometer (Beckman Coulter, Krefeld, Germany). B-cell subsets were identified by multicolor staining using IgD, CD10, CD19, CD20, CD21, CD24, CD27, CD38, CD45, CD80, CD86 and HLA-DR (BD,NJ USA). Phenotypic characterization of T-cell subsets was performed using CD3, CD4, CD8, CD25, CD45, CD45RA, CCR7, CD127 and CD161 (BD, NJ USA). The data was analyzed using the Kaluza Software (Version 1.1, Beckman Coulter, Krefeld, Germany).

### Immunohistochemistry

Immunohistochemistry on paraffin-embedded tumor samples was performed with an automated stainer (Medac) at the institute of pathology, Hildegardis-Hospital-Cologne. CD79a, CD20, Ki67, CD8 and IgG-kappa (Dako, Hamburg, Germany) antibodies were used for immunohistochemistry.

### Nephelometry of IgG subclasses

Immunoglobulin-G-subclasses (IgG1, IgG2, IgG3 and IgG4) were analyzed on a Siemens BN ProSpec Analyzer using a Human IgG-Subclass liquid reagent kit (Binding Site, Birmingham, UK) according to the companies' instructions.

### Statistical analysis

Statistical analyses were performed with SPSS Version 20 (IBM corp.) and GraphPad Prism 5 (Graphpad Software, Inc.). Normal distribution was tested using the Shapiro-Wilk test of normality. In case of normal distribution student's T-test was performed otherwise significance was analyzed by the Mann-Whitney-U-test.

## SUPPLEMENTARY TABLES


