# Supplementary material for: Characterization of tumor-associated B-cell subsets in patients with colorectal cancer
Source: Oncotarget. 2014 May 9;5(13):4651–64. doi: 10.18632/oncotarget.1701 (PMC4148088; doi:10.18632/oncotarget.1701)
Supplement: Supplementary file 1 [file oncotarget-05-4651-s001.pdf]

SUPPLEMENTARY TABLE

Table 1: Pathological characteristics of included patients

Pat. No	Sample	Localisation	UICC	pT	pN	G	L	V	M	(R)CTx
1	T,PB	Colon	St. II	3	0	2	1	0	0	no
2	T,PB	Colon	St. III	3	1	2	0	0	0	colon
3	T,PB	Rectum	St. IV	3	2	2	1	0	1	no
4	T,PB	Colon	St. IV	3	2	3	1	0	1	colon
5	T,PB	Colon	St. III	3	2	2	0	0	0	colon
6	T,PB	Colon	St. IV	3	2	2	0	0	1	colon
7	T,PB	Colon	St. II	4	0	3	1	0	0	colon
8	T,PB	Rectum	St. II	3	0	2	0	0	0	no
9	T,PB	Colon	St. III	3	1	2	0	0	0	colon
10	T,PB	Rectum	St. I	2	0	2	0	0	0	no
11	T,PB	Colon	St. IV	3	2	3	1	0	1	no
12	T,PB	Colon	St. III	3	1	2	0	0	0	colon
13	T,PB	Colon	St. III	3	1	2	1	0	0	colon
14	T,PB	Colon	St. IV	3	0	2	0	0	1	colon
15	T,PB	Colon	St. II	3	0	2	0	0	0	colon
16	T,PB	Colon	St. II	3	0	2	0	0	0	colon
17	T,PB	Rectum	St. II	3	0	2	0	0	0	no
18	T,PB	Rectum	St. III	3	1	2	0	0	0	no
19	T,PB	Colon	St. III	3	1	2	0	0	0	colon
20	T,PB	Colon	St. III	3	1	2	0	0	0	colon
21	PB	Rectum	St. III	3	2	2	0	0	0	yes
22	PB	Rectum	St. II	3	0	2	0	0	0	no
23	T,PB	Colon	St. IV	4	2	3	1	1	1	colon
24	T,PB	Colon	St. II	4	0	3	1	0	0	colon
25	PB	Colon	St. II	3	0	2	0	0	0	colon
26	T,PB	Colon	St. II	3	0	2	0	0	0	colon
27	T,PB	Colon	St. II	3	0	2	0	0	0	colon
28	T,PB	Rectum	St. IV	3	2	2	0	0	1	no
29	T,PB	Colon	St. II	3	0	2	0	0	0	colon
30	PB	Colon	St. IV	3	1	2	0	0	1	colon
31	T,PB	Colon	St. III	3	2	2	1	0	0	colon
32	PB	Colon	St. IV	3	2	2	1	0	1	colon
33	T,PB	Colon	St. II	3	0	2	0	0	0	colon
34	T,PB	Rectum	St. II	3	0	2	0	0	0	no

(*Continued*)

Pat. No	Sample	Localisation	UICC	pT	pN	G	L	V	M	(R)CTx
35	T,PB	Rectum	St. III	3	2	2	1	0	0	no
36	T,PB	Rectum	St. IV	3	2	2	1	1	1	no
37	T,PB	Colon	St. I	2	0	2	0	0	0	colon
38	T,PB	Rectum	St. II	3	0	2	0	0	0	no
39	T,PB	Colon	St. III	3	1	2	0	0	0	colon
40	T,PB	Rectum	St. IV	4	1	2	0	1	1	no
41	PB	Rectum	St. IV	3	2	2	1	0	1	no
42	T,PB	Colon	St. III	2	1	2	1	0	0	colon
43	M,PB	Colon	St. IV	-	-	2	-	-	1	colon
44	M,PB	Colon	St. IV	3	1	2	1	0	1	colon
45	PB	Colon	St. II	3	0	2	0	0	0	colon
46	T	Colon	St. II	3	0	2	0	0	0	colon
47	T,PB	Colon	St. III	3	1	3	1	0	0	colon
48	M	Colon	St. IV	-	-	-	-	-	1	metast.
49	M	Rectum	St. IV	-	-	-	-	-	1	metast.
50	M	Rectum	St. IV	-	-	-	-	-	1	metast.
51	M	Rectum	St. IV	-	-	-	-	-	1	metast.

Sample: PB= peripheral blood; T= tumor sample; M= sample from colorectal liver metastasis
